# Supplementary material for: Comparative genomics provides new insights into the diversity, physiology, and sexuality of the only industrially exploited tremellomycete: Phaffia rhodozyma
Source: BMC Genomics. 2016 Nov 9;17:901. doi: 10.1186/s12864-016-3244-7 (PMC5103461; doi:10.1186/s12864-016-3244-7)
Supplement: Additional file 6: — List of orphan genes with links to PFAM (related to Additional file 1: Table S1). (ZIP 1428 kb) [file 12864_2016_3244_MOESM6_ESM.zip › BLAST_HTML_FTR/G00685_P.html]

BLAST Search Results


```
BLASTP 2.2.27+


Reference:
Stephen F. Altschul, Thomas L. Madden, Alejandro A. Schäffer,
Jinghui Zhang, Zheng Zhang, Webb Miller, and David J. Lipman (1997),
"Gapped BLAST and PSI-BLAST: a new generation of protein database
search programs", Nucleic Acids Res. 25:3389-3402.


Reference for
composition-based statistics:
Alejandro A. Schäffer, L. Aravind, Thomas L. Madden, Sergei
Shavirin, John L. Spouge, Yuri I. Wolf, Eugene V. Koonin, and
Stephen F. Altschul (2001), "Improving the accuracy of PSI-BLAST
protein database searches with composition-based statistics and
other refinements", Nucleic Acids Res. 29:2994-3005.


Database: nr
           71,551,133 sequences; 26,053,659,533 total letters


Query= G00685_P

Length=439
                                                                      Score     E
Sequences producing significant alignments:                          (Bits)  Value

emb|CED85170.1|  hypothetical protein [Xanthophyllomyces dendrorh...   902    0.0  
emb|CCA71373.1|  hypothetical protein PIIN_05312 [Piriformospora ...  43.9    0.28 
emb|CDH60517.1|  predicted protein [Lichtheimia corymbifera JMRC:...  39.7    4.9  


 >emb|CED85170.1| hypothetical protein [Xanthophyllomyces dendrorhous]
Length=438

 Score =  902 bits (2332),  Expect = 0.0, Method: Compositional matrix adjust.
 Identities = 438/438 (100%), Positives = 438/438 (100%), Gaps = 0/438 (0%)

Query  1    MDTYVIILADLLGLDRGVFTAKPWRKFIKRRVLIHMKARFGLCSPLQTSFSHPYVPTETS  60
            MDTYVIILADLLGLDRGVFTAKPWRKFIKRRVLIHMKARFGLCSPLQTSFSHPYVPTETS
Sbjct  1    MDTYVIILADLLGLDRGVFTAKPWRKFIKRRVLIHMKARFGLCSPLQTSFSHPYVPTETS  60

Query  61   LCTLNPSEPGSPVSRPSTDSPSIGSEPTPDPSDRSFLLATPCRARSSSLTVQTTIPSCSR  120
            LCTLNPSEPGSPVSRPSTDSPSIGSEPTPDPSDRSFLLATPCRARSSSLTVQTTIPSCSR
Sbjct  61   LCTLNPSEPGSPVSRPSTDSPSIGSEPTPDPSDRSFLLATPCRARSSSLTVQTTIPSCSR  120

Query  121  IVKTEPIQTDSVNPSDPTSSCLSHSDIQQAIQRARRSEPRDHDLDASVPIKRTEARVQNI  180
            IVKTEPIQTDSVNPSDPTSSCLSHSDIQQAIQRARRSEPRDHDLDASVPIKRTEARVQNI
Sbjct  121  IVKTEPIQTDSVNPSDPTSSCLSHSDIQQAIQRARRSEPRDHDLDASVPIKRTEARVQNI  180

Query  181  ILDVCRRMGQIYPELTINNTEEDYEGDVKVKISEIKRGLEGSNEVSGSDHDYDSDDGDDS  240
            ILDVCRRMGQIYPELTINNTEEDYEGDVKVKISEIKRGLEGSNEVSGSDHDYDSDDGDDS
Sbjct  181  ILDVCRRMGQIYPELTINNTEEDYEGDVKVKISEIKRGLEGSNEVSGSDHDYDSDDGDDS  240

Query  241  EDEGYKGDHGQNRTFSPRKTRSDKRKLQDMSVSEEQDRRRFIKTQWTLETRPKTRTCTTQ  300
            EDEGYKGDHGQNRTFSPRKTRSDKRKLQDMSVSEEQDRRRFIKTQWTLETRPKTRTCTTQ
Sbjct  241  EDEGYKGDHGQNRTFSPRKTRSDKRKLQDMSVSEEQDRRRFIKTQWTLETRPKTRTCTTQ  300

Query  301  EEIRQLKRLIREVGVRKNWGREFRGRELDFKWQLGYLQTIIHGVKTQQPPYTVQMYQKLR  360
            EEIRQLKRLIREVGVRKNWGREFRGRELDFKWQLGYLQTIIHGVKTQQPPYTVQMYQKLR
Sbjct  301  EEIRQLKRLIREVGVRKNWGREFRGRELDFKWQLGYLQTIIHGVKTQQPPYTVQMYQKLR  360

Query  361  SSELLARKAEHVDTPTELEGSILNRSASELSLSLRAGHAGPEKAMNVKSWRGWRYIMVDD  420
            SSELLARKAEHVDTPTELEGSILNRSASELSLSLRAGHAGPEKAMNVKSWRGWRYIMVDD
Sbjct  361  SSELLARKAEHVDTPTELEGSILNRSASELSLSLRAGHAGPEKAMNVKSWRGWRYIMVDD  420

Query  421  EGNELPDAIWEAPVRPFL  438
            EGNELPDAIWEAPVRPFL
Sbjct  421  EGNELPDAIWEAPVRPFL  438


>emb|CCA71373.1| hypothetical protein PIIN_05312 [Piriformospora indica DSM 11827]
Length=372

 Score = 43.9 bits (102),  Expect = 0.28, Method: Compositional matrix adjust.
 Identities = 17/47 (36%), Positives = 30/47 (64%), Gaps = 0/47 (0%)

Query  298  TTQEEIRQLKRLIREVGVRKNWGREFRGRELDFKWQLGYLQTIIHGV  344
            + +E++++LK  +   GVRK W +EF G E + K Q+  L+ I+ G+
Sbjct  241  SNEEQVKRLKSFVVACGVRKKWAKEFEGIEDNPKKQISRLKEILEGL  287


>emb|CDH60517.1| predicted protein [Lichtheimia corymbifera JMRC:FSU:9682]
Length=317

 Score = 39.7 bits (91),  Expect = 4.9, Method: Compositional matrix adjust.
 Identities = 27/93 (29%), Positives = 51/93 (55%), Gaps = 2/93 (2%)

Query  289  ETRPKTRTCTTQEEIRQLKRLIREVGVRKNWGREFRGRELDFKWQLGYLQTIIHGVKTQQ  348
            E++ + +T  T+E I++LK  I + GVRK W +E    +   K Q+  L+ I+  +  + 
Sbjct  175  ESKKEHKTSATEESIKRLKMYINKCGVRKVWQKELADCKT-MKDQVEKLKKILADLGVEG  233

Query  349  PPYTVQMYQKLRSSELLARKAEHVDTPTELEGS  381
             P T++  QK+++   +  + E +DT   +E S
Sbjct  234  RP-TLEKCQKVKAEREIKAEVESLDTENIIESS  265


Lambda      K        H        a         alpha
   0.316    0.133    0.396    0.792     4.96 

Gapped
Lambda      K        H        a         alpha    sigma
   0.267   0.0410    0.140     1.90     42.6     43.6 

Effective search space used: 4284913739535


  Database: nr
    Posted date:  Sep 23, 2015 12:05 AM
  Number of letters in database: 26,053,659,533
  Number of sequences in database:  71,551,133


Matrix: BLOSUM62
Gap Penalties: Existence: 11, Extension: 1
Neighboring words threshold: 11
Window for multiple hits: 40
```
